# Chimeric nanoparticles for targeting mitochondria in cancer cells†

**DOI:** 10.1039/d1na00644d

**Published:** 2022-01-18

**Authors:** Aman Bajpai, Nakshi Nayan Desai, Shalini Pandey, Chinmayee Shukla, Bhaskar Datta, Sudipta Basu

**Affiliations:** Discipline of Chemistry, Indian Institute of Technology (IIT) Gandhinagar Palaj Gandhinagar Gujarat 382355 India bdatta@iitgn.ac.in sudipta.basu@iitgn.ac.in; Discipline of Biological Engineering, Indian Institute of Technology (IIT) Gandhinagar Palaj Gandhinagar Gujarat 382355 India

## Abstract

Mitochondrial dysfunction is implicated in myriad diseases, including cancer. Subsequently, targeting mitochondrial DNA (mt-DNA) in cancer cells has emerged as an unorthodox strategy for anti-cancer therapy. However, approaches targeting only one component of the mitochondrial “central dogma” can be evaded by cancer cells through various mechanisms. To address this, herein, we have engineered mitochondria-targeting cholesterol-based chimeric nanoparticles (mt-CNPs) consisting of cisplatin, camptothecin, and tigecycline, which can simultaneously impair mt-DNA, mitochondrial topoisomerase I (mt-Top1), and mitochondrial ribosomes. mt-CNPs were characterized as being positively charged, spherical in shape, and 187 nm in diameter. Confocal microscopy confirmed that mt-CNPs efficiently localized into the mitochondria of A549 lung cancer cells within 6 h, followed by mitochondrial morphology damage and the subsequent generation of reactive oxygen species (ROS). mt-CNPs showed remarkable cancer-cell killing abilities compared to free-drug combinations in A549 (lung), HeLa (cervical), and MCF7 (breast) cancer cells. These mitochondria-targeting lipidic chimeric nanoparticles could be explored further to impair multiple targets in mitochondria, helping researchers to gain an understanding of mitochondrial translational and transcriptional machinery and to develop new strategies for cancer therapy.

## Abstract 2

Mitochondrial dysfunction is implicated in myriad diseases, including cancer.

## Graphical abstract

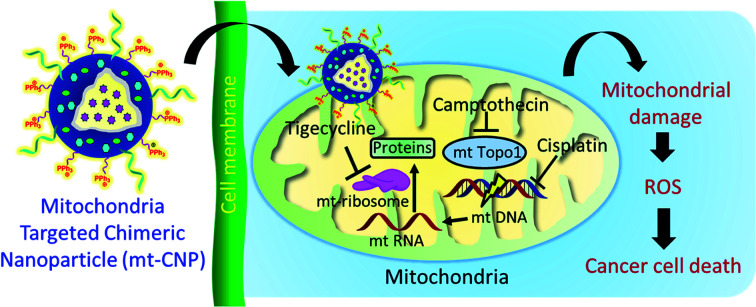

## Introduction

In recent years, mitochondria have gained immense recognition in disease-related biomedical research due to their role in numerous significant biological phenomena, including metabolism, biosynthesis, cell survival/death programming, signalling pathways, and so on.^1–4^ Consequently, targeting and perturbing mitochondrial functionality in a diseased state like cancer has emerged as a novel therapeutic strategy.^5–7^ Fascinatingly, mitochondria contain their own set of DNA, RNA, and ribosomes for synthesizing OXPHOS-associated proteins through conserved mitochondrial transcription and translational pathways.^8–10^ Hence, impairing mitochondrial “central dogma” in relation to the routing of small molecules has been found to help improve therapeutic outcomes and overcome drug resistance.^11,12^ However, the selective targeting of mitochondria in the cellular milieu of cancer cells is still a daunting challenge, although nanoscale materials can be effectively explored to achieve this.^13–16^

Due to their vast potential as drug delivery vehicles, nanomaterials have been explored as a way to deliver DNA-damaging drugs into the mitochondria of cancer cells for improved therapeutic efficacy.^17–22^ However, mitochondrial machinery for the repair of DNA damage can evade the effects of nanoparticle-mediated mitochondrial DNA damage.^23,24^ As a result, it is crucial to impair multiple mitochondrial transcriptional and translational components simultaneously to obtain enhanced effects. Towards this end, we recently developed α-tocopheryl succinate-based Cerberus nanoparticles and graphene-oxide-based self-assembled nanoparticles for targeting mitochondrial DNA and mitochondrial topoisomerase I in cancer cells.^25,26^ Nevertheless, targeting only the mitochondrial translational components in cancer cells without perturbing the transcriptional machinery will eventually lead to the emergence of drug resistance. Hence, we hypothesize that mitochondrial transcriptional and translational mechanisms should be impaired simultaneously to upgrade therapeutic outcomes.

To achieve this, herein we have engineered mitochondria-targeting cholesterol-based chimeric nanoparticles (mt-CNPs), consisting of cisplatin, camptothecin, and tigecycline, for the synchronized perturbation of mitochondrial DNA (mt-DNA), mitochondrial topoisomerase I (mt-Top1), and mitochondrial ribosomes (mt-ribosomes) in cancer cells. These spherical positively charged mt-CNPs successfully homed in on the mitochondria of A549 lung cancer cells within 3 h, leading to mitochondrial morphology disruption followed by the generation of reactive oxygen species (ROS). This mt-CNP-mediated mitochondrial damage triggered a remarkable enhancement in A549 and HeLa cancer cell death compared to the use of free drug combinations, and there was a much lower level of non-cancerous human embryonic kidney cell (HEK293) death. We anticipate that the nanoparticle-mediated simultaneous impairment of mitochondrial transcriptional and translational machinery in cancer cells will provide a new understanding of mitochondrial biology, supporting novel anti-cancer therapy approaches.

## Results and discussion

### Synthesis of cholesterol–drug conjugates

**Fig. 1:**
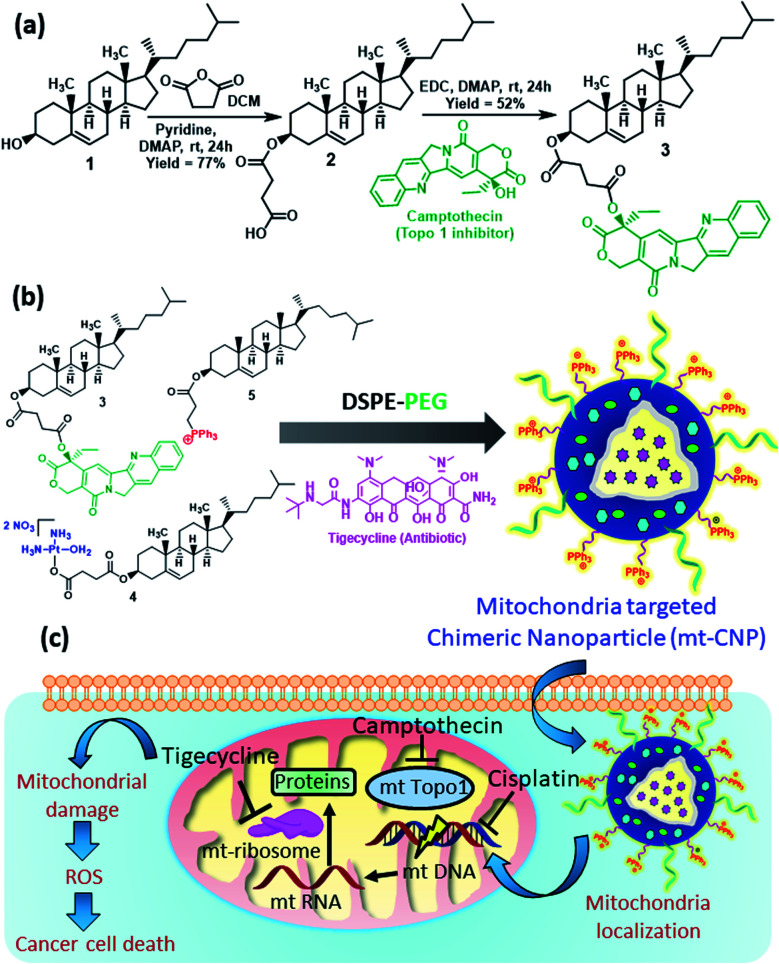
(a) The synthesis of the cholesterol–camptothecin conjugate (3). (b) The synthesis of mitochondria-targeting chimeric nanoparticle (mt-CNPs). (c) A schematic representation of the mechanism of action of mt-CNPs in cancer cells.

Cholesterol was chosen for the development of mitochondria-targeting chimeric nanoparticles due to its biocompatibility and as it is one of the major components of cell membranes. Cholesterol (1) was first converted to a cholesterol–succinic acid conjugate (2) in the presence of succinic anhydride and pyridine (Fig. 1a).^27^ Camptothecin, a topoisomerase-I inhibitor (FDA approved drug), was reacted with cholesterol–succinic acid in the presence of EDC and DMAP as coupling agents to obtain the cholesterol–camptothecin conjugate (3) in 52% yield. The cholesterol–camptothecin conjugate was characterized *via*^1^H-NMR, ^13^C-NMR, and HR-MS spectroscopy (Fig. S1–S3, ESI†). We have also synthesized a cholesterol–cisplatin conjugate (4) and cholesterol–triphenylphosphine conjugate (5) using previously reported methods and these were characterized *via* standard spectroscopic techniques.^27,28^

### Engineering mitochondria-targeting chimeric nanoparticles

We engineered mitochondria-targeting chimeric nanoparticles (mt-CNPs) using the cholesterol conjugates (3, 4, and 5), phosphatidylcholine (PC), DSPE–PEG, and tigecycline (which was encapsulated) in a 1 : 1 : 6 : 0.6 : 1 : 1 ratio *via* a solvent evaporation–hydration–extrusion method (Fig. 1b).^27^ We have incorporated a cholesterol–triphenylphosphine conjugate (5) to target mitochondria, as the positively charged triphenylphosphine (TPP) moiety localizes the nanoparticles at sub-cellular mitochondria.^28^ The loading of cisplatin, camptothecin, and tigecycline was quantified based on UV-vis spectroscopy (Fig. S4a, ESI†). The loading levels of tigecycline, camptothecin, and cisplatin were calculated to be 418 μg mL^−1^, 368 μg mL^−1^, and 190 μg mL^−1^, respectively (Fig. S4b, ESI†). We also calculated the drug loading efficiencies to be 42%, 37%, and 19% for tigecycline, camptothecin, and cisplatin, respectively.

**Fig. 2:**
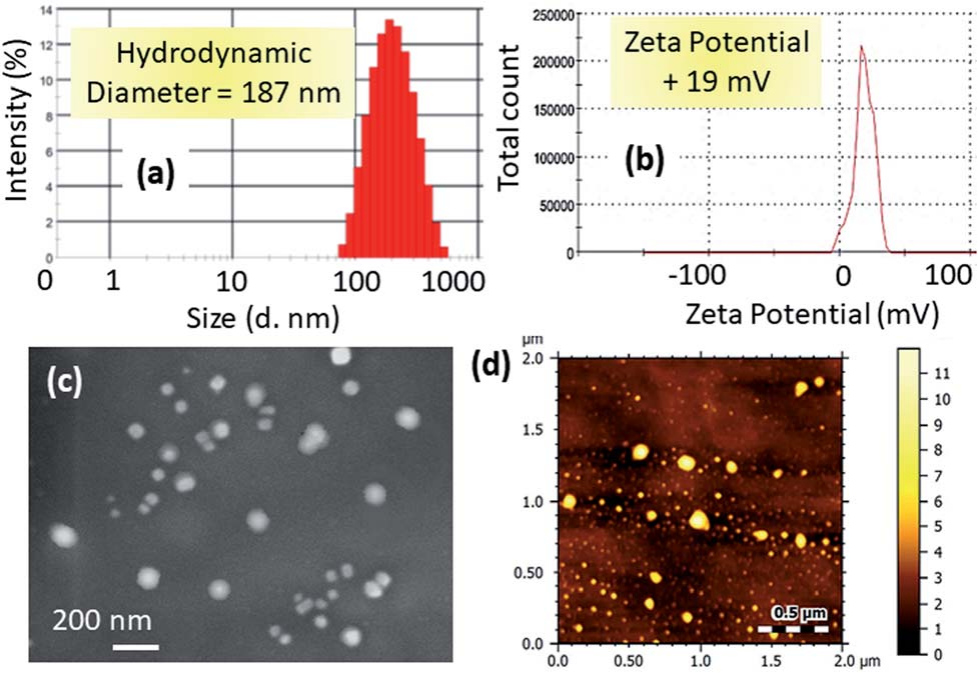
The (a) hydrodynamic diameter and (b) surface charge of mt-CNPs based on dynamic light scattering (DLS) measurements. (c) SEM and (d) AFM images of mt-CNPs.

We characterized the hydrodynamic diameter and surface charge of mt-CNPs *via* dynamic light scattering (DLS). The hydrodynamic diameter of mt-CNPs was found to be 187 nm (Fig. 2a). For successful homing in on mitochondria, mt-CNPs should possess highly positive charge.^29,30^ The surface charge of mt-CNPs was found to be +19 mV, which is appropriate for mitochondria localization (Fig. 2b).

We further visualized the morphology of mt-CNPs *via* electron microscopy. FESEM and AFM images (Fig. 2c and d) demonstrated that mt-CNPs were spherical in shape and less than 200 nm in diameter. From light scattering and electron microscopy analysis, it was evident that mt-CNPs have positive surface charge with a spherical shape and a sub-200 nm diameter.

To be successfully applied clinically, mt-CNPs should be stable for a long time during storage and in a biological environment. To evaluate the stability, we incubated mt-CNPs in water at 4 °C (storage conditions), in phosphate-buffered saline (PBS) at 37 °C (physiological conditions) and in DMEM cell culture medium at 37 °C (biological conditions) for 7 days. We checked the size of mt-CNPs *via* DLS. We observed that the size of mt-CNPs in water at 4 °C changed from 185 nm to 122 nm over 7 days (Fig. S5a, ESI†). On the other hand, mt-CNPs changed in size from 185 nm to 159 nm and 152 nm in PBS and DMEM, respectively, at 37 °C after 7 days (Fig. S5b and c, ESI†). These stability assays indicated that these mt-CNPs are relatively stable in physiological and biological environments over 7 days and under storage conditions for nearly 4 days.

### mt-CNPs localized into mitochondria

**Fig. 3:**
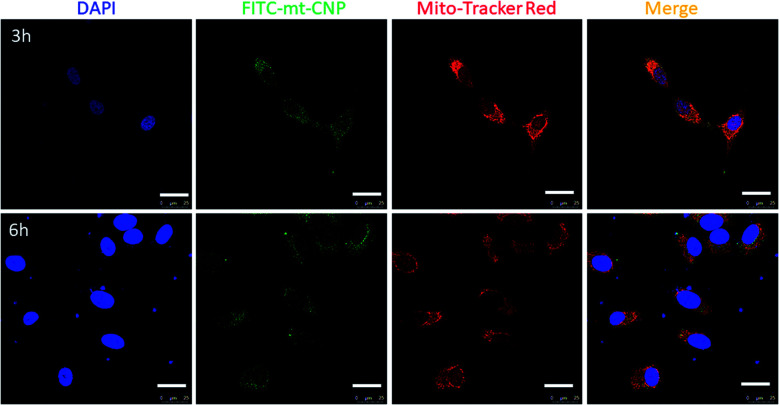
Confocal laser scanning microscopy images of A549 cells treated with FITC–mt-CNPs (green) after 3 h and 6 h. Mitochondria and nuclei were stained with MitoTracker Red (red) and DAPI (blue), respectively. Scale bar = 25 μm.

Due to the positive surface charge, we expected mt-CNPs to be localized in the mitochondria of cancer cells (Fig. 1c). To validate our hypothesis, we synthesized mt-CNPs encapsulating fluorescein isothiocyanate (FITC), a green fluorescent dye, (FITC–mt-CNP) for tracking the sub-cellular localization. We have chosen A549 lung cancer cells for visualization due to their enlarged mitochondria and functional clustered mitochondrial DNA nucleoids.^31^ We treated A549 lung cancer cells with FITC–mt-CNPs for 3 h and 6 h, followed by co-staining with MitoTracker Red and DAPI. Cellular mitochondria were stained with MitoTracker Red dye while the nuclei of cells were stained with DAPI (blue fluorescence) dye. The fixed cells were then visualized *via* confocal microscopy. Fig. 3 demonstrates that the green fluorescent FITC–mt-CNPs were localized into the mitochondria within 3 h, producing overlapping yellow fluorescence signals. Moreover, from confocal imaging, it was also seen that FITC–mt-CNPs were retained in the mitochondria for 6 h (Fig. 3 and S6, ESI†). Interestingly, after 12 h, the green fluorescence signal of FITC was also found to localize in the nucleus and outside the mitochondria. We also found that a low-level FITC signal persisted in the mitochondria after 12 h. This observation can be explained as arising because within 12 h mt-CNPs would disrupt mitochondria partially, leading to the ejection of encapsulated FITC from mitochondria followed by its spatial distribution in cytosol and nuclei (Fig. S6, ESI†). These confocal images demonstrated that FITC–mt-CNPs can be localized in mitochondria within 3 h, be retained there for over 6 h, and partially damage mitochondria within 12 h.

After localization into sub-cellular mitochondria, mt-CNPs should release drugs to effectively inhibit the mitochondrial targets. It is highly challenging to isolate the mitochondrial matrix in order to evaluate the drug release profile from mt-CNPs. However, the pH of the mitochondrial matrix is alkaline (a pH value of ∼8) due to proton efflux from the matrix for ATP synthesis.^32–35^ Hence, we evaluated the release of drugs from mt-CNPs at alkaline pH. We incubated mt-CNPs in buffer at pH 8 in a time-dependent manner and evaluated the drug release using UV-vis spectroscopy at *λ*_max_ values of 706 nm, 361 nm, and 271 nm for cisplatin, camptothecin, and tigecycline, respectively. It was found that after 72 h, 45%, 56%, and 49% of tigecycline, camptothecin, and cisplatin, respectively, were released from mt-CNPs (Fig. S7a, ESI†). As a control, we also quantified the release of drugs from mt-CNPs at physiological pH (7.4) and lysosomal pH (5.5) in a time-dependent manner. We observed that at pH 7.4, even after 72 h, only 29% tigecycline, 32% camptothecin, and 33% cisplatin was released (Fig. S7b, ESI†). On the other hand, at pH = 5.5, 41% tigecycline, 13% camptothecin, and 17% cisplatin were released after 72 h (Fig. S7c, ESI†). From these confocal images and drug release assays, it was evident that mt-CNPs were indeed localized into the mitochondria of A549 cells and released drugs in a slow and time- and pH-dependent manner.

### mt-CNP induces mitochondrial damage

**Fig. 4:**
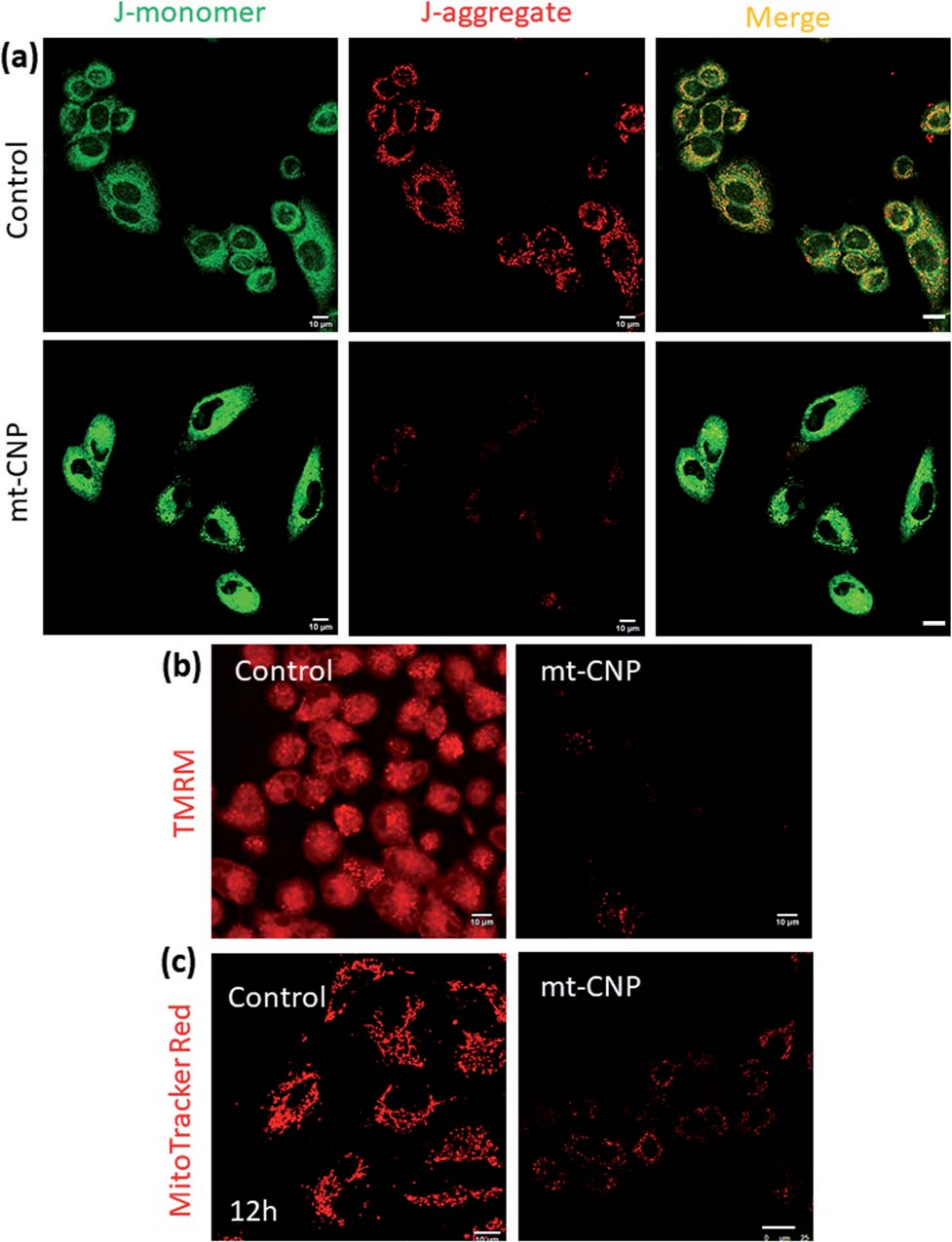
Confocal microscopy images of A549 cells treated with mt-CNPs followed by (a) JC1 staining, (b) staining with red fluorescent TMRM to evaluate mitochondrial depolarization, and (c) staining with MitoTracker Red to visualize the mitochondrial morphology after 12 h. Scale bars = 10 μm.

After localizing into sub-cellular mitochondria and carrying out drug release, mt-CNPs should damage mitochondria *via* the simultaneous impairment of mt-DNA, mt-ribosomes, and mt-Top-1. To evaluate the mitochondrial impairment, we investigated the mitochondrial membrane potential *via* JC1 assays. The cationic dye JC1 can adopt a monomeric (green fluorescence, *λ*_max_ = 525 nm) form and an aggregated (red fluorescence, *λ*_max_ = 590 nm) form. In healthy mitochondria-containing cells, JC1 remains in monomeric and aggregated forms outside and inside mitochondria. However, in damaged mitochondria, aggregated JC1 is released from mitochondria to cytosol in monomeric form, leading to a change from red fluorescence to green. Hence, we treated A549 cells with mt-CNPs for 24 h, then incubated the cells with JC1 dye and visualized the cells under confocal microscopy. The microscopy images showed that in the case of the control cells, both green and red fluorescence signals remained with equal intensity, leading to yellow fluorescence (J-monomer : J-aggregate = 1) (Fig. 4a). However, in mt-CNP-treated cells, we observed a remarkable decrease in the red fluorescence intensity along with an increase in the green fluorescence intensity (J-monomer : J-aggregate = 3.5). These JC1 assays clearly confirmed that mt-CNPs triggered mitochondrial membrane depolarization significantly compared to the control case.

To further evaluate the mitochondrial damage, we treated A549 cells with mt-CNPs for 24 h, followed by incubation with tetramethylrhodamine methyl ester (TMRM), a red fluorescent dye for staining healthy undamaged mitochondria. TMRM is effluxed from cells when the mitochondria are damaged. We visualized the cells using confocal microscopy. Fig. 4b demonstrates that non-treated control cells retained red fluorescence signals from TMRM from healthy mitochondria. Interestingly, treatment with a free-drug combination also resulted in high levels of red fluorescence from TMRM from A549 cells, indicating marginal mitochondrial damage (Fig. S8, ESI†) However, mt-CNP-treated cells showed a remarkable reduction in the TMRM signal, confirming mitochondrial damage and the subsequent efflux of TMRM from the cells.

We further visualized the mitochondrial morphology after treatment with mt-CNPs. We treated A549 cells with mt-CNPs for 12 h and 24 h, followed by staining mitochondria with MitoTracker Red fluorescent dye. The cells were then visualized *via* confocal microscopy. The confocal images (Fig. 4c) revealed that the non-treated control cells retained the characteristic fibrous and thread-like morphology of healthy mitochondria after 12 h (Fig. 4c) and 24 h (Fig. S9, ESI†). However, mt-CNPs disrupted the elongated filamentous mitochondrial morphology and produced small fragmented punctate structures after 12 h and 24 h. Moreover, mt-CNPs damaged the mitochondrial morphology in a time-dependent manner. Interestingly, we observed traces of filamentous morphology after 12 h of treatment with mt-CNPs (Fig. S10, ESI†), showing that some undamaged mitochondria existed. We anticipate that, even within 12 h, all three drugs started to be released inside the mitochondria, damaging the mitochondrial morphology partially; the exact mechanism is yet to be explored. However, no such morphology was observed after 24 h of treatment with mt-CNPs (Fig. S10, ESI†), confirming complete mitochondrial damage. As a control, we treated A549 cells with a free-drug combination and visualized the mitochondrial morphology. We observed a significant level of filamentous mitochondrial morphology, confirming marginal mitochondrial damage in the presence of the free-drug combination (Fig. S11, ESI†). These confocal imaging studies clearly confirmed the mitochondrial morphology impairment due to mt-CNPs. This result is also in agreement with our previous study.^26^

**Fig. 5:**
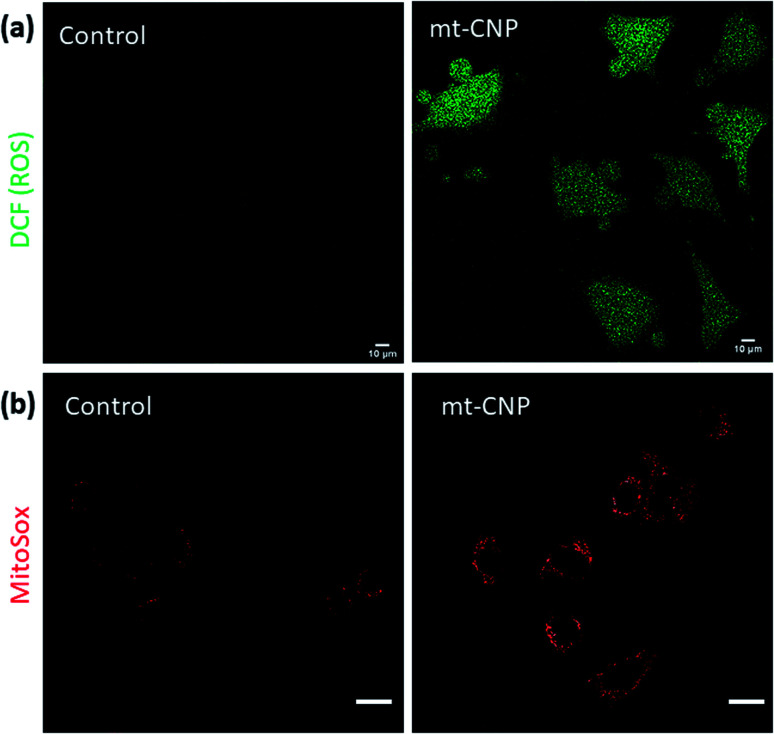
Confocal microscopy images of A549 cells treated with mt-CNPs followed by staining with (a) H_2_DCFDA dye and (b) MitoSox Red dye to observe reactive oxygen species (ROS). Scale bars = 10 μm.

Mitochondrial damage leads to an increase in the levels of sub-cellular reactive oxygen species (ROS).^36^ To evaluate the sub-cellular ROS generation, we performed H_2_DCFDA assays. H_2_DCFDA is a non-fluorescent dye which reacts with ROS inside cells to produce a green fluorescent DCF dye. We treated A549 cells with mt-CNPs for 24 h, followed by incubation with H_2_DCFDA. The cells were visualized under confocal microscopy. The microscopy images (Fig. 5a and S12, ESI†) showed that non-treated cells and cells treated with a free-drug combination hardly showed any ROS generation, as confirmed by the negligible levels of green fluorescence. However, mt-CNP-treated cells demonstrated a significant increase in green fluorescence due to the generation of sub-cellular ROS. To further evaluate the generation of superoxides, we treated A549 cells with mt-CNPs for 24 h and incubated the cells with MitoSox dye which produces a red fluorescence signal in the presence of superoxides. Confocal microscopy images (Fig. 5b and S13, ESI†) showed that untreated A549 cells hardly produced any ROS. On the other hand, mt-CNP treatment remarkably increased the red fluorescence signal, confirming the production of ROS. These confocal microscopy images evidently confirmed that mt-CNPs damaged mitochondria and triggered superoxide generation.

**Fig. 6:**
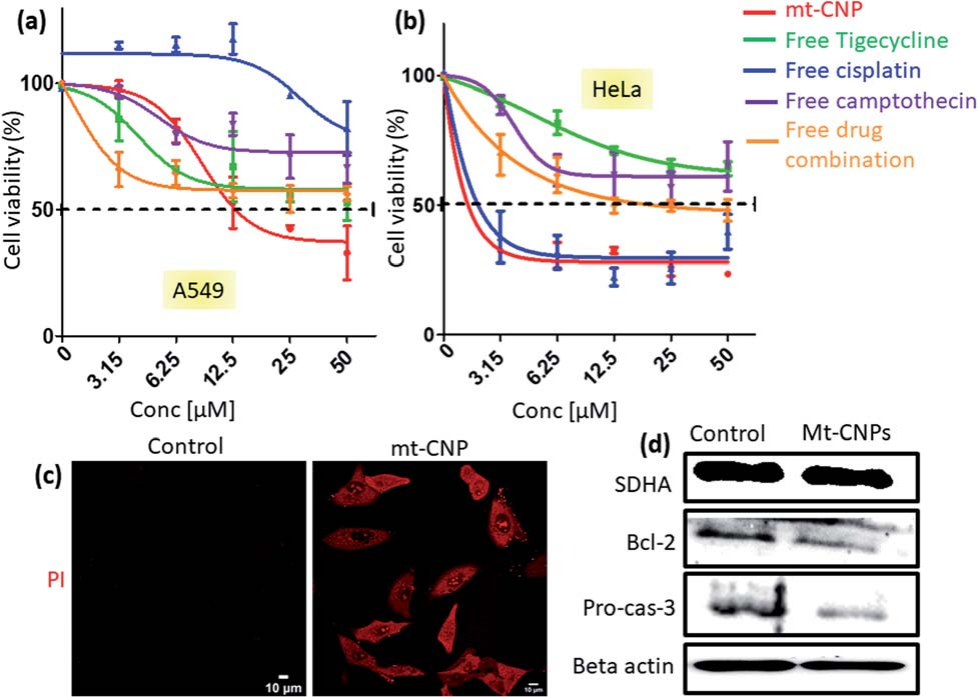
MTT assays showing the viabilities of (a) A549 and (b) HeLa cells in response to treatment with mt-CNPs and free drugs in a dose dependent manner 24 h post-incubation. (c) Confocal microscopy images of A549 cells treated with mt-CNPs for 24 h, followed by staining with PI; scale bars = 10 μm. (d) Western blot analysis of SDHA, Bcl-2, and pro-caspase-3 in A549 cells after treatment with mt-CNPs for 24 h.

In our previous study, we have shown that mitochondria-targeting nanoparticles can impair mitochondrial DNA, mitochondrial topoisomerase I, and mitochondrial ribosomes.^25,28^ To further prove that nuclear DNA remained intact, we evaluated the expression of succinate dehydrogenase subunit A (SDHA) *via* western blot analysis. It was observed that SDHA expression remained unchanged after treatment with mt-CNPs compared to the non-treated control cells (Fig. 6d).

### Cell death and apoptosis

Mitochondrial damage due to mt-CNPs would lead to cell death through apoptosis. To evaluate the cell death efficacy, we treated A549 cells with mt-CNPs in a dose-dependent manner for 24 h, and cell viabilities were measured *via* MTT assays. As controls, we also treated A549 cells with free tigecycline, free cisplatin, free camptothecin, and a combination of the free drugs at an analogous ratio to what was present in mt-CNPs. We found that mt-CNPs showed remarkable A549 cell killing abilities with IC_50_ = 13.2 μM (Fig. 6a). On the other hand, neither the free drugs nor the drug combination killed over 50% of A549 cells, even at concentrations of 50 μM. We also evaluated the efficacy of mt-CNPs toward HeLa cervical cancer cells 24 h post-incubation. We have chosen HeLa cells as the mitochondrial content remains constant in HeLa cells even during the cell cycle and division.^37^ Interestingly, in HeLa cells, mt-CNP showed much improved cell killing efficacy, with an IC_50_ value of 2 μM (Fig. 6b). On the contrary, the treatment with the free-drug combination showed a much higher IC_50_ value of 12.5 μM. Moreover, free cisplatin also showed similar efficacy, with an IC_50_ value of 2.3 μM. Free tigecycline and camptothecin demonstrated marginal cell killing effects, even at high concentrations of 50 μM. We further assessed the efficacy of mt-CNPs towards MCF7 breast cancer cells after 24 h. Free tigecycline and drug combination treatment showed low cell killing effects, with IC_50_ values > 50 μM (Fig. S14a, ESI†). On the other hand, free cisplatin and free camptothecin showed IC_50_ values of 2.5 and 8.5 μM, respectively. However, mt-CNPs demonstrated much higher efficacy in killing MCF7 cells, with an IC_50_ value of 1.1 μM. To further evaluate the effects of mt-CNPs on non-cancerous cells, we treated HEK293 human embryonic kidney cells with mt-CNPs and free tigecycline in a dose-dependent manner over 24 h and quantified the cell viabilities *via* MTT assays. We observed that free tigecycline showed HEK293 cell killing effects, with an IC_50_ value of 7.2 μM (Fig. S14b, ESI†). Interestingly, mt-CNPs showed nearly 50% cell killing only at a concentration of 50 μM concentration, with a much higher IC_50_ value of 26.1 μM (Fig. S14, ESI†). These MTT assays confirmed that mt-CNPs remarkably killed A549 and HeLa cells in a much-improved manner compared to free drugs and combination therapy. Furthermore, mt-CNPs showed much less toxicity in HEK293 cells compared with free tigecycline alone.

Apoptosis is one of the possible mechanisms for cell death through mitochondrial damage. We have evaluated apoptosis in A549 cells through propidium iodide (PI) assays, as PI can enter late apoptotic cells and bind with nuclear DNA. We treated A549 cells with mt-CNPs for 24 h, followed by staining the cells with red fluorescent PI, and they were then visualized under confocal microscopy. Fluorescent microscopy images of the non-treated control cells hardly showed any red fluorescent signals, confirming that apoptosis was not induced (Fig. 6c). However, the mt-CNP-treated cells showed a remarkable increase in the level of red fluorescence signals inside the cells compared to the control cells, evidently confirming the induction of apoptosis in A549 cells. Furthermore, we also evaluated the induction of apoptosis *via* western blot analysis. We treated A549 cells with mt-CNPs for 24 h and evaluated the expression of antiapoptotic Bcl-2 and effector caspase 3 cleavage *via* western blot. We observed that mt-CNPs reduced the expression of antiapoptotic Bcl-2 as well as caspase-3 significantly compared to the control cells (Fig. 6d). Furthermore, we also performed the Caspase-Glo® 3/7 assay, which is a homogeneous and luminescent assay that measures caspase-3 and -7 activities, which are markers of apoptosis. We treated A549 cells with mt-CNPs for 24 h and incubated them with the Caspase-Glo® 3/7 reagents, followed by measuring the fluorescence at *λ*_max_ = 488 nm. The fluorescence intensity of the mt-CNP-treated cells increased 2-fold compared with the control cells (Fig. S15, ESI†). These confocal microscopy, gel electrophoresis, and Caspase-Glo® 3/7 studies clearly confirmed that mt-CNPs induced cell death through mitochondrial damage *via* triggering apoptosis.

## Conclusions

In conclusion, we have engineered cholesterol-based mitochondria-targeting chimeric nanoparticles (mt-CNPs) comprising cisplatin, camptothecin, and tigecycline to impair mitochondrial DNA, topoisomerase I, and ribosomes simultaneously. These spherical, positively charged, sub-200 nm particles localized efficiently into the mitochondria of A549 cells within 6 h, where they induced mitochondrial damage and the generation of reactive oxygen species (ROS). Finally, mt-CNPs showed remarkable lung cancer, cervical cancer, and breast cancer cell killing abilities compared to free-drug combinations. We anticipate that these mitochondria-targeting chimeric nanoparticles could evolve into an interestingly tool to impair multiple targets in mitochondria, helping researchers to gain an understand of mitochondrial biology and to develop next-generation cancer therapeutics.

## Experimental section

### Synthesis of the cholesterol–CPT conjugate (3)

Conjugate 2 (100 mg, 0.229 mmol) was dissolved in 3 mL of DMF, and 1-ethyl-3-(3-dimethylaminopropyl)carbodiimide (EDC) (43 mg, 0.229 mmol) and 4-dimethylaminopyridine (DMAP) (28 mg, 0.229 mmol) were added, followed by stirring for 15 min at 0 °C. The mixture was then bought to room temperature, and camptothecin (40 mg, 0.114 mmol) was added to the mixture. The resulting solution was kept under nitrogen for 12 h at room temperature. The reaction was monitored *via* TLC and the solution was filtered using Whatman filter paper. After removing the organic solvent under reduced pressure, the obtained crude product was purified using silica gel column chromatography with 15% ethyl acetate/hexane to obtain the pure compound 3. Yield = 52%. ^1^H NMR (500 MHz, CDCl_3_): *δ* (ppm) 8.38 (s, 1H), 8.24 (d, *J* = 5 Hz, 1H), 7.92 (d, *J* = 5 Hz, 1H), 7.82 (m, 1H), 7.69–7.63 (m, 1H), 7.32 (s, 1H), 5.67 (d, *J* = 15 Hz, 1H), 5.39 (d, *J* = 15 Hz, 1H), 5.28 (d, *J* = 5.0 Hz, 2H), 5.04 (d, *J* = 5.0 Hz, 1H), 4.57–4.48 (m, 1H), 2.92–2.75 (m, 2H), 2.71–2.54 (m, 4H), 2.33–2.22 (m, 2H), 2.19–2.12 (m, 3H), 1.97 (m, 2H), 1.87–1.77 (m, 4H), 1.65 (m, 1H), 1.52 (m, 3H), 1.33 (m, 7H), 1.18–1.05 (m, 8H), 1.02–0.96 (m, 7H), 0.91 (d, *J* = 5.0 Hz, 6H), 0.88–0.84 (m, 12H). ^13^C NMR (126 MHz, CDCl_3_): *δ* (ppm) 171.26, 171.04, 167.28, 157.30, 152.23, 148.77, 145.99, 145.93, 139.34, 130.98, 130.50, 129.68, 128.37, 128.04, 127.86, 122.57, 122.18, 120, 96.50, 76.04, 74.33, 66.89, 56.56, 56.06, 49.84, 49.68, 42.16, 39.60, 39.39, 37.72, 36.64, 36.27, 36.07, 35.65, 31.68, 31.59, 29.32, 29.07, 28.86, 28.09, 27.88, 27.47, 24.12, 23.72, 22.69, 22.43, 21.79, 19.00, 18.60, 11.68, 7.47. HR-MS: *m*/*z* for C_51_H_64_N_2_O_7_^+^[M]+, calculated = 817.08; observed = 817.47.

## Author contributions

AB, NND, SP, and CS performed all the experiments and collected the data. BD and SB wrote the manuscript.

## Conflicts of interest

There are no conflicts to declare.

## Supplementary Material

NA-004-D1NA00644D-s001
