# Supplementary material for: Chimeric nanoparticles for targeting mitochondria in cancer cells
Source: Nanoscale Adv. 2022 Jan 18;4(4):1112–8. doi: 10.1039/d1na00644d (PMC9419202; doi:10.1039/d1na00644d)
Supplement: NA-004-D1NA00644D-s001 [file NA-004-D1NA00644D-s001.pdf]

Electronic Supplementary Material (ESI) for Nanoscale Advances.
This journal is © The Royal Society of Chemistry 2022

Supporting Information

Chimeric Nanoparticle for Targeting Mitochondria in Cancer Cells

Aman Bajpai,[a] *Nakshi Nayan Desai,*[b] *Shalini Pandey,*[a] *Chinmayee Shukla,*[b] *Bhaskar Datta,**[ab] *Sudipta Basu**[a]

a Discipline of Chemistry, Indian Institute of Technology (IIT) Gandhinagar, Palaj, Gandhinagar, Gujarat, 382355, India

b Discipline of Biological Engineering, Indian Institute of Technology (IIT) Gandhinagar, Palaj, Gandhinagar, Gujarat, 382355, India

* Corresponding authors.

Email: Sudipta.basu@iitgn.ac.in and bdatta@iitgn.ac.in

Materials:

Materials: Cholesterol, camptothecin, succinic anhydride, cis-platin, 3-bromopropionyl chloride, triphenylphosphine, silver nitrate, pyridine, 1-ethyl-3-(3-dimethylaminopropyl)carbodiimide, 4-dimethylaminopyridine (DMAP), sodium iodide, acetonitrile, L-α-phosphatidylcholine (PC), 1,2-distearoyl-*sn*-glycero-3–phosphoethanolamine-N-[amino(polythylene glycol)2000] (DSPE-PEG), Sephadex G-25, tetramethylrhodamine methyl ester (TMRM), sodium dodecyl sulfate (SDS), MitoSOX™ red mitochondrial superoxide indicator for live-cell imaging, silicon wafer for field emission scanning electron microscopy (FESEM) were procured from Sigma-Aldrich, handheld extruder kit (including 0.2 nm whatman nucleopore track-etch membrane, whatman filter supports and 1.0 mL hamiltonian syringes) were purchased from Avanti Polar Lipids Inc. Dulbecco's modified eagle's medium (DMEM) and 3-(4,5-dimethylthiazol-2-yl)-2,5-diphenyltetrazolium bromide (MTT) were bought from Sigma-Aldrich. HeLa and A549 cells were obtained from National Centre for Cell Science, Pune, Cells were cultured in DMEM + 10% FBS (for HeLa) and DMEM F-12K + 10% FBS (for A549) at 37°C in 5% CO_2 under humidified conditions.

Methods:

Synthesis and characterization of Cholesterol-CPT conjugate (3).

Conjugate 2 (100 mg, 0.229 mmol) was dissolved in 3 ml DMF, to the solution 1-ethyl-3-(3-dimethylaminopropyl) carbodiimide (EDC) (43 mg, 0.229 mmol) and 4-dimethylaminopyridine (DMAP) (28 mg, 0.229 mmol) were added and stirred for 15 minutes at 0 °C. The mixture was then bought to the room temperature and camptothecin (40 mg, 0.114 mmol) was added to the mixture. The resulting solution was kept under nitrogen condition for 12 h at room temperature. The reaction was monitored by TLC and was filtered using Whatmann filter paper. After removing the solvent under reduced pressure, the obtained crude product was purified using silica gel column chromatography with 15% ethyl acetate/hexane to obtain the pure compound 3. (Yield= 56%). ^{1}H NMR (500 MHz, CDCl3) δ 8.38 (s, 1H), 8.24 (d, J = 5 Hz, 1H), 7.92 (d, J = 5 Hz, 1H), 7.82 (m, 1H), 7.69 – 7.63 (m, 1H), 7.32 (s, 1H), 5.67 (d, J = 15 Hz, 1H), 5.39 (d, J = 15 Hz, 1H), 5.28 (d, J = 5.0 Hz, 2H), 5.04 (d, J = 5.0 Hz, 1H), 4.57 – 4.48 (m, 1H), 2.92 – 2.75 (m, 2H), 2.71 – 2.54 (m, 4H), 2.33 – 2.22 (m, 2H), 2.19 – 2.12 (m, 3H), 1.97 (m, 2H), 1.87 – 1.77 (m, 4H), 1.65 (m, 1H), 1.52 (m, 3H), 1.33 (m, 7H), 1.18 – 1.05 (m, 8H), 1.02 – 0.96 (m, 7H), 0.91 (d, J = 5.0 Hz, 6H), 0.88 – 0.84 (m, 12H). ^{13}C NMR (126 MHz, CDCl3) δ 171.26, 171.04, 167.28, 157.30, 152.23, 148.77, 145.99, 145.93, 139.34, 130.98, 130.50, 129.68, 128.37, 128.04, 127.86, 122.57, 122.18, 120, 96.50, 76.04, 74.33, 66.89, 56.56, 56.06, 49.84, 49.68, 42.16, 39.60, 39.39, 37.72, 36.64, 36.27, 36.07, 35.65, 31.68, 31.59, 29.32, 29.07, 28.86, 28.09, 27.88, 27.47, 24.12, 23.72, 22.69, 22.43, 21.79, 19.00, 18.60, 11.68, 7.47. HR-MS: m/z: for $C_{51}H_{64}N_2O_7^+$[M]+: calculated= 817.08, observed= 817.47.

Synthesis of mt-CNPs.

The mitochondria targeted chimeric nanoparticles were synthesized by mixing 1 mg each of cholesterol conjugate 3, 4 and 5 with 1 mg tigecycline in DCM. To the mixture, L-α-phosphatidyl choline (PC) and 1,2-distearoyl–sn-phosphoethanolamine-N- [amino (polyethylene glycol)-2000] (DSPE-PEG2000) were added in a ratio of 6: 0.6 with respect to tigecycline. Evaporation of the solvent using rotary evaporator resulted in thin lipid-film, followed by drying under vacuum for 30 minutes. Further 1 mL milli q water was added to it, the hydrated mixture was rotated at low pressure for 2 hours at 60 °C with frequent sonication. The solution was then passed through Sephadex G-25 column. Solution obtained was then extruded through 200 nm filter.

Loading of drug in mt-CNPs.

The amount of drug loading was calculated by plotting a calibration graph for the tigecycline, camptothecin and o-Phenylenediamine conjugated cis-platin in the concentration range of 1.25 to 50 µM in water. The absorbance was measured at 271 nm, 361 nm, and 707 nm for tigecycline, camptothecin, and o-Phenylenediamine conjugated cis-platin respectively. A linear graph was plotted for absorbance (A) *vs*. concentration (C). The drug loading in the

nanoparticle was evaluated by UV at λ_{max} = 271, 361, and 707 nm. Drug loading in NPs was calculated from the calibration graph in triplicate sets. Drug loading efficiency was calculated using the equations as given below:

drug loading efficiency= {amount of drug loaded into nanoparticle/ amount of drug used}x 100%

Size, shape, surface charge and stability determination.

The size, shape, surface charge, morphology, and stability of CHM-Tig NPs were evaluated by dynamic light scattering (DLS), zeta potential, field-emission scanning electron (FESEM), and atomic force microscopy (AFM). The samples for the DLS and zeta were prepared by adding 20 μl of stock solution of NPs (1mg/mL) by dispersing in 1 mL of water. The stability studies were performed by DLS using water and DMEM as dispersion media. The samples for the DLS and zeta were measured on Malvern-Nano-ZS. All the SEM and AFM samples were prepared by drop-casting method on the silicon wafer. SEM and AFM were recorded on JOEL-JSM7600F and Multimode 8(Bruker) respectively.

In Vitro Assays.

Mitochondrial internalization by confocal imaging, cell viability by MTT assay, mitochondrial membrane permeabilization by Tetramethylrhodamine, methyl ester (TMRM) and mitochondrial ROS detection using Mito-SOX were performed.

Cell culture:

HeLa and A549 cells were obtained from National Centre for Cell Science, Pune. Cells were cultured in DMEM + 10% FBS (for HeLa) and DMEM F-12K + 10% FBS (for A549) at 37°C in 5% CO_2 under humidified conditions.

Cell Viability Assay.

MTT assay was performed for cell viability detection. $5*10^3$ HeLa and A549 cells were treated with CHM-Tig NPs for 24 hours. 3-(4,5-dimethylthiazol-2-yl)-2,5-diphenyltetrazolium bromide (MTT, Sigma-Aldrich, M5655) was added to the wells for 4 hours to allow the formation of formazan crystals which were further solubilized in a solubilization buffer containing 10% SDS in 0.01 M HCl. The absorbance was recorded at 570 nm using a spectrophotometer. The cell viability percentage was calculated by considering the untreated cells as 100% viability and the efficiency of Tigecycline encapsulated CHM-Tig NPs were compared with free drugs. Experiments were repeated thrice.

Mitochondria Localization.

A quantity of 1.5×10^4 A549 cells were seeded in a Lab Tek chamber slide 8 well (per well). After 12 h attachment, cells were incubated with CHM-FITC NPs for 3h and 6h time point. Mitochondria were labeled with MitoTracker Red FM and imaged by CLSM.

JC1 Assay.

A quantity of 1.5×10^4 A549 cells were seeded in a Lab Tek chamber slide 8 well (per well). Mt-CNPs were incubated into the cells for 24 h. A control was kept without adding the nanoparticle. Cells were then washed twice with PBS (pH 7.4) and incubated with JC1 dye (10 μg mL−1) at 37 °C for 20 min. Before adding JC1 dye, cells were visualized using CLSM at 590 nm fluorescence emission wavelength, and the red fluorescence signal obtained from the nanoparticles were calculated to be deducted from JC1 fluorescence emission signal as a baseline correction. The JC1 dyes in cells were visualized and quantified by CLSM.

Mito-sox assay.

Seed $1 \text{ X } 10^4$ cells per well in a 35mm glass bottom cell culture plate. After 24 hrs on day1, the cells were treated with IC50 concentration of drug and CHM-Tig NPs. On day 2, the media was aspirated and the cells were washed twice with PBS. Add 1 mL of 5 μM MitoSOX™ reagent working solution (prepared in HBSS solution) to cover cells

adhering to coverslip(s). Incubate cells for 10 minutes at 37˚C, protected from light. Wash cells gently three times with warm buffer. Obtained the live cell imaging using confocal microscopy that enable visualization of ROS generation in mitochondria.

PI Assay.

An amount of 5 × 10^4 A549 cells were seeded in Nunc glass bottom dishes and incubated overnight in a 5% CO_2 incubator at 37 °C. The cells were then treated with mt-CNPs at IC_{50} concentration, while the control plate was left untreated. After 24 h, the compounds were aspirated, and plates were washed gently with PBS. Propidium iodide (Thermo Fisher, catalog number P1304MP) was dissolved in PBS and was added to a final concentration of 500 nM, and plates were incubated for 10 min. The bright field and confocal images were captured using a Leica confocal laser scanning microscope using the 63× oil immersion objective and at 561 nm wavelength.

Caspase 3/7 assay.

The caspase 3/7 assay is to detect caspase-3 and -7 activities in cell culture. 20 x 10^3 cells were seeded per well in 96 well plate. Seeded cells were then treated with mt-CNPs and a mixture of drugs with the same concentration of mt-CNPs at IC50, incubate for 24 h. The freshly prepared caspase 3/7 reagent is added in 1:1 ratio of media and reagent, keep the plate for 30 minutes of incubation at 37˚C, protected from light. Take the fluorescent reading at 488 nm absorbance wavelength using MTT plate reader.

Wester Blotting.

After 24 hours of treatment with mt-CNPs, cells were lysed with RIPA buffer. 30 µg of protein was loaded and separated on SDS-PAGE gel and transferred to a 0.22 µm nitrocellulose membrane (Millipore Corporation). The membrane was blocked with 5% skimmed milk in TBST and subsequently probed with primary and secondary antibodies. The blots were developed with Clarity Western ECL substrate (Bio-rad, 1705061) and quantified using ImageJ software after normalization to β-actin.

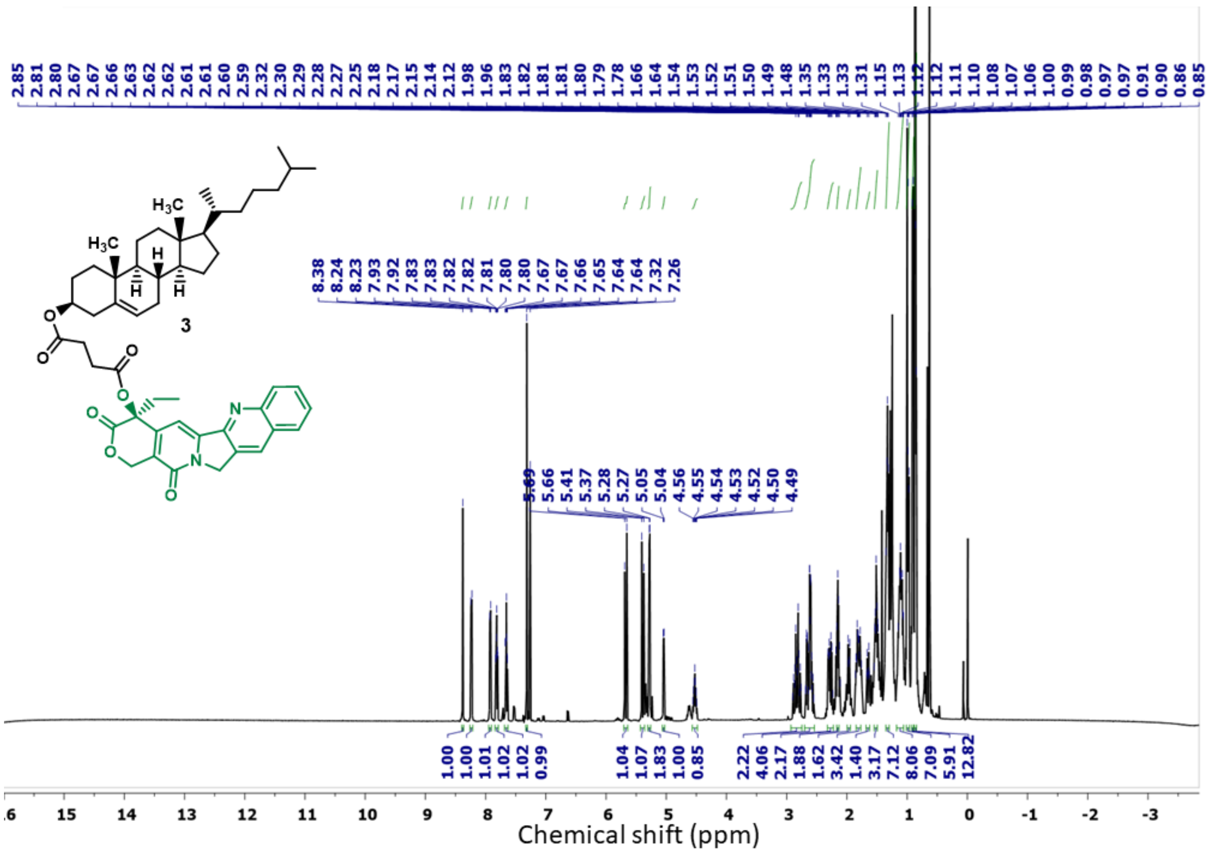

Fig. S1: ^{1}H NMR spectra of cholesterol-camptothecin conjugate (**3**).

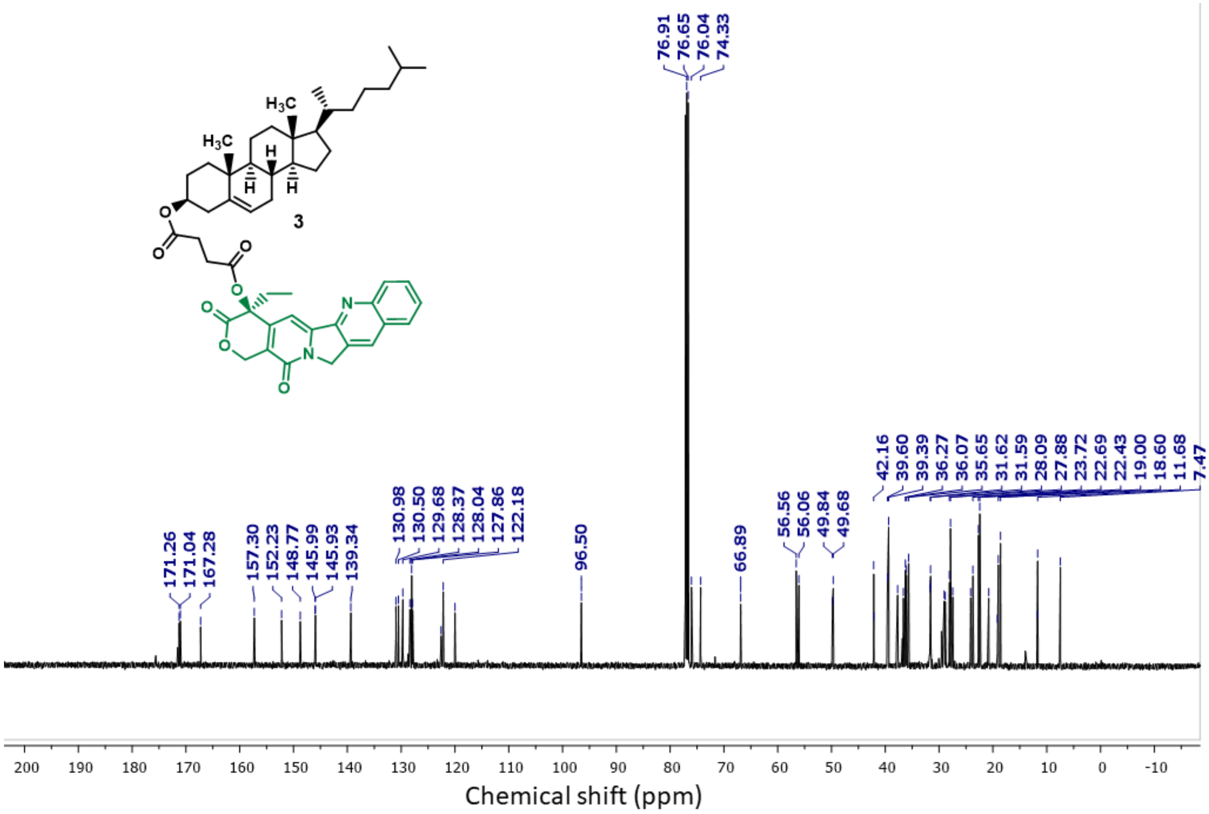

Fig. S2: ^{13}C NMR spectra of cholesterol-camptothecin conjugate (**3**).

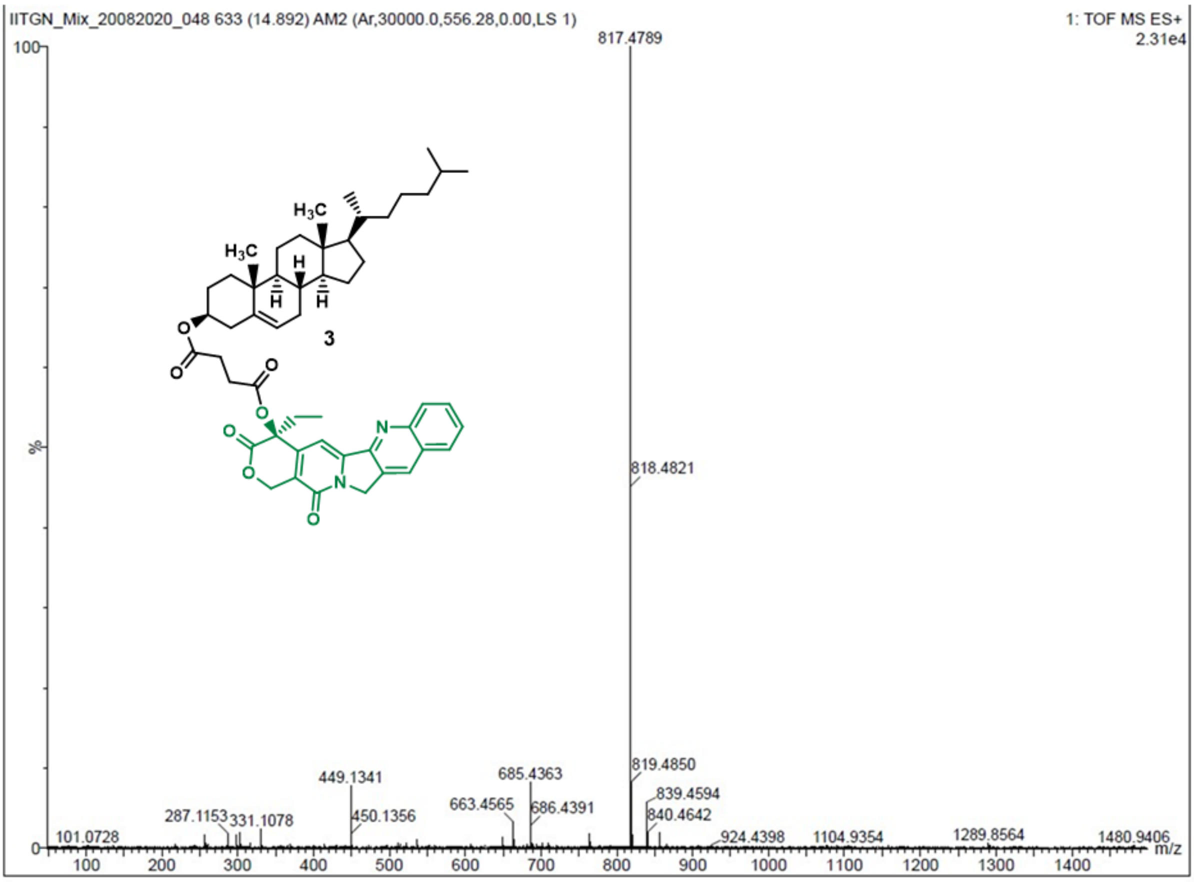

Fig. S3: HR-MS spectra of cholesterol-camptothecin conjugate (**3**).

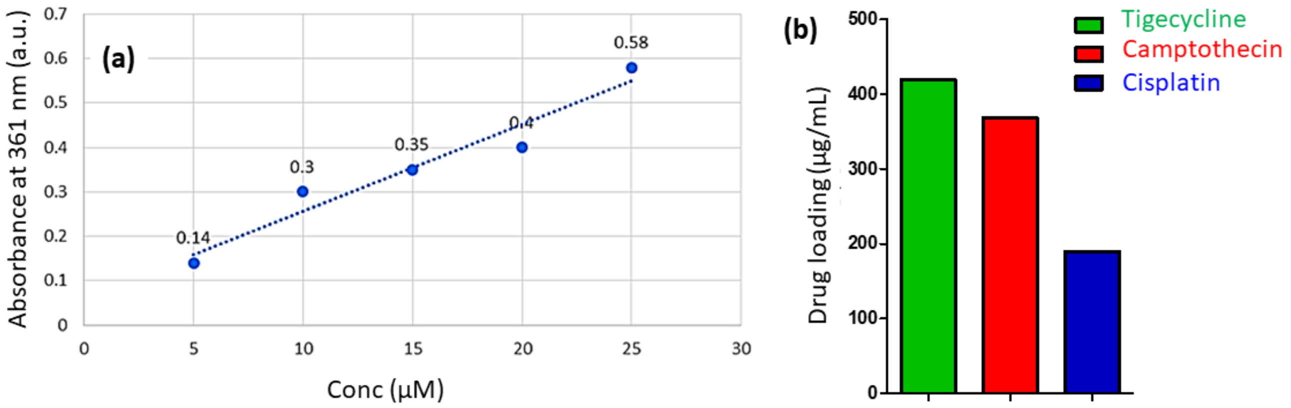

Fig. S4: (a) Absorbance versus concentration calibration graph for camptothecin at λmax = 361 nm from UV-Vis spectroscopy. (b) Loading of cisplatin, camptothecin and tigecycline in mt-CNPs quantified from UV-Vis spectroscopy.

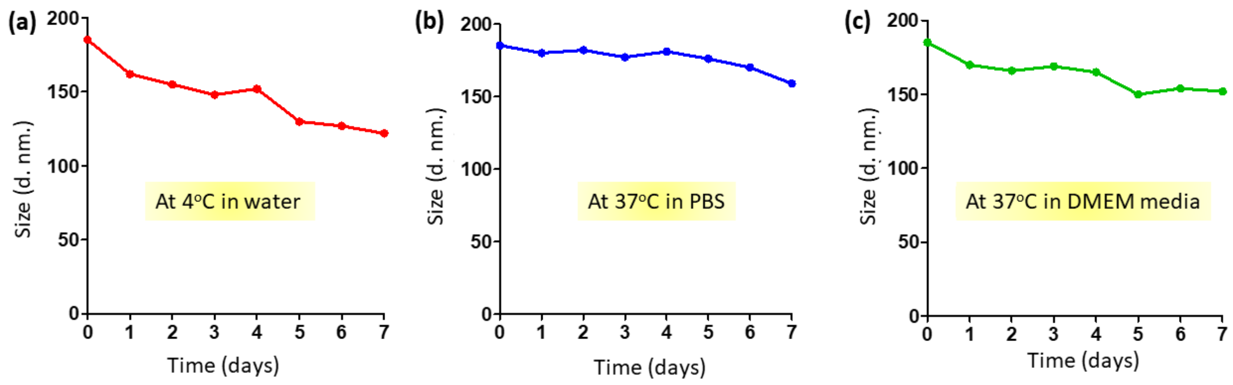

Fig. S5. Stability of mt-CNPs determined by DLS at 4°C in water, (b) 37°C in PBS and (c) 37°C in DMEM cell culture media over 7 days.

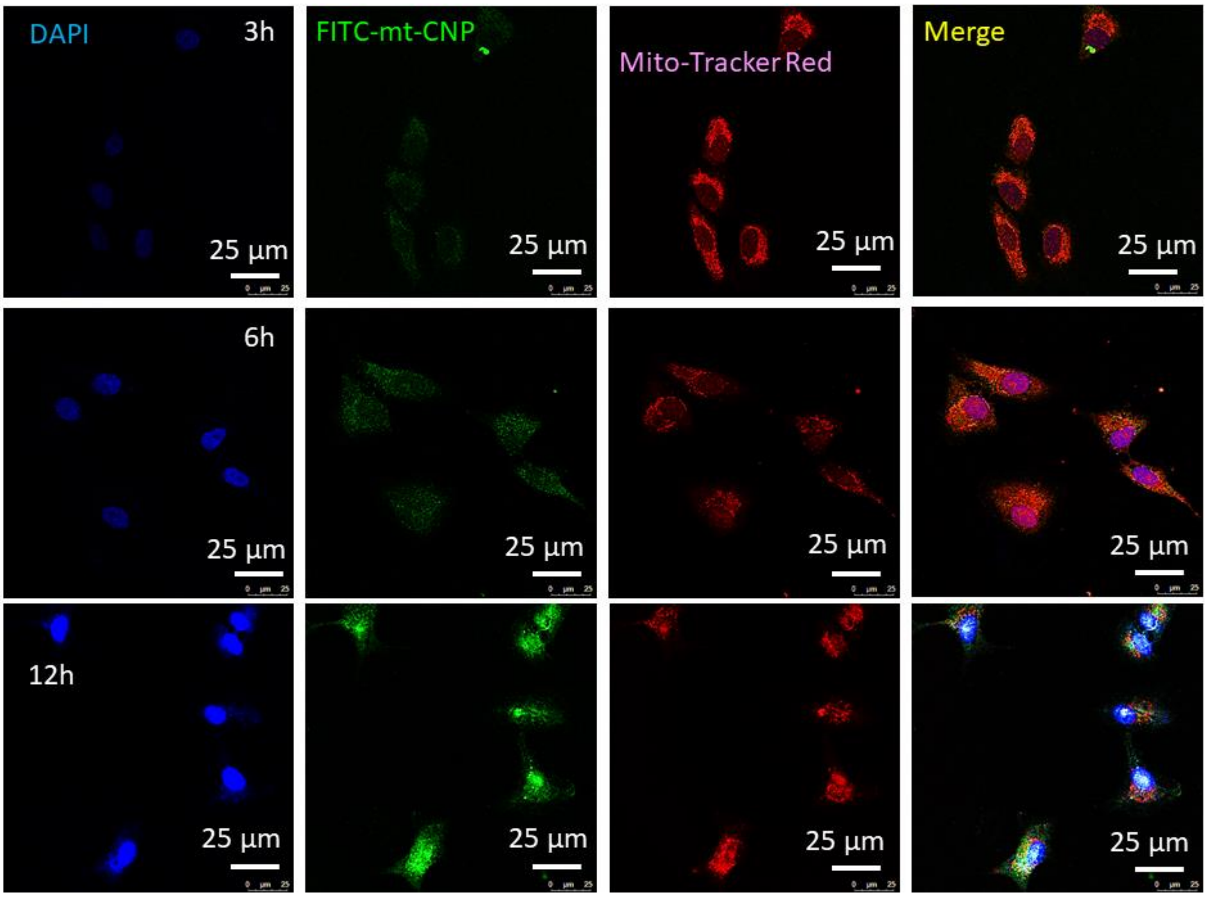

Fig. S6. Confocal microscopy images of A549 cells after treatment with FITC-mt-CNP (green) at 3h, 6h and 12h. Mitochondria and nucleus were stained with MitoTracker Red (red) and DAPI (blue) respectively. Scale bar = 25 μm.

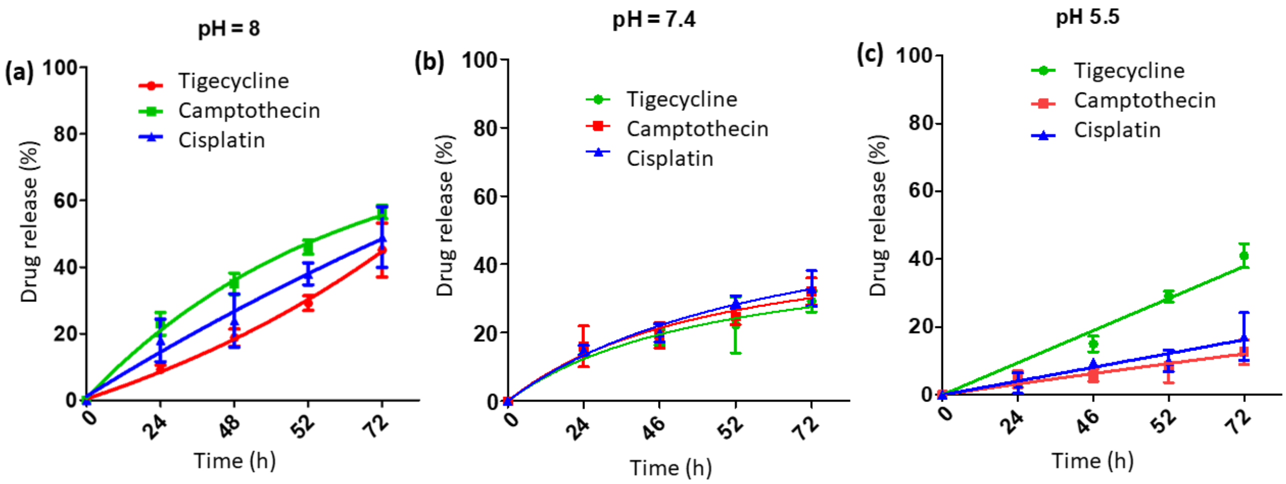

Fig. S7: Release of drugs from mt-CNPs in a time dependent manner over 72h at (a) pH = 8, (b) 7.4 and (c) pH = 5.5.

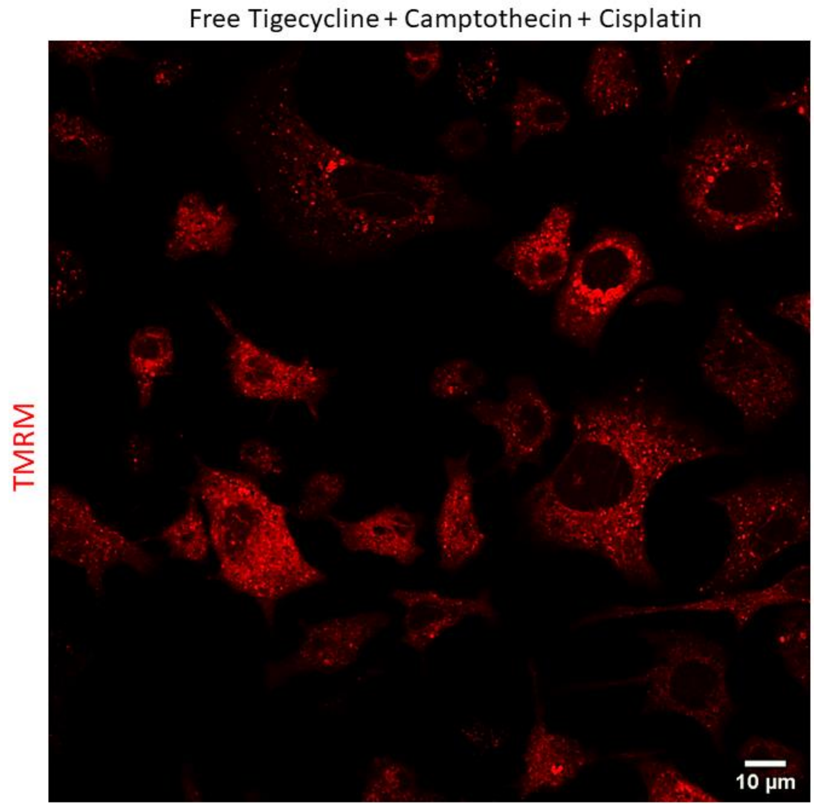

Fig. S8: Confocal microscopy images of A549 cells treated with free drug combinations followed by staining with red fluorescent TMRM to evaluate mitochondrial depolarization. Scale bar = 10 µm.

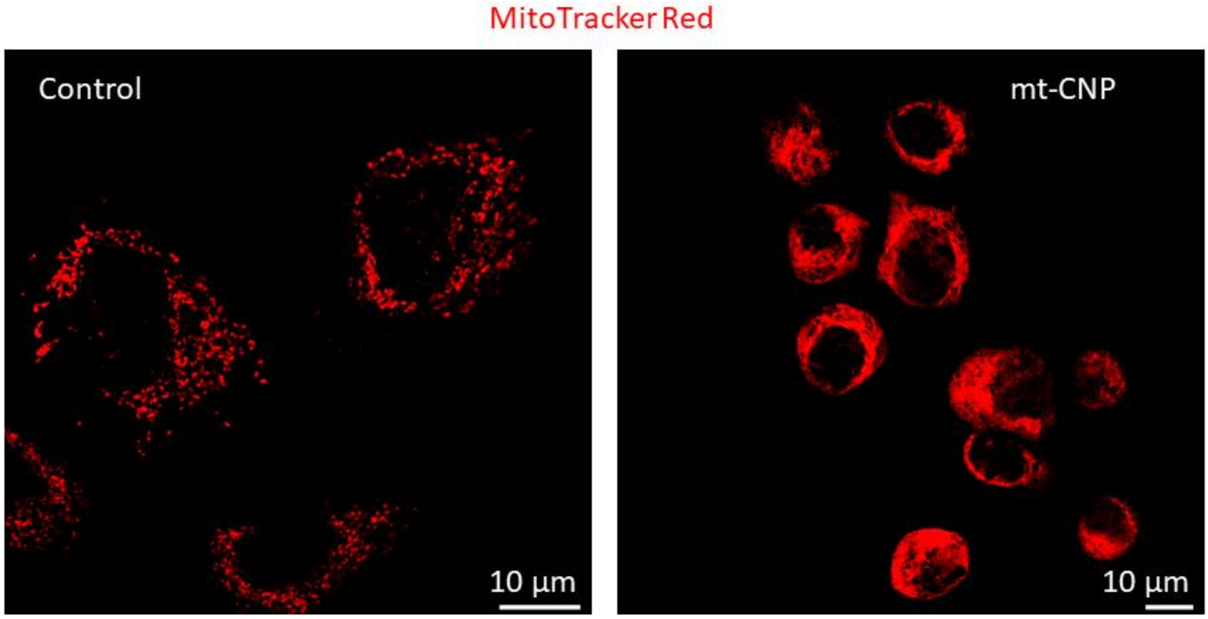

Fig. S9: Confocal microscopy images of A549 cells after treatment with mt-CNPs for 24h followed by staining the mitochondria with MitoTracker Red to observe mitochondrial morphology at 24h. Scale bar = 10 µm.

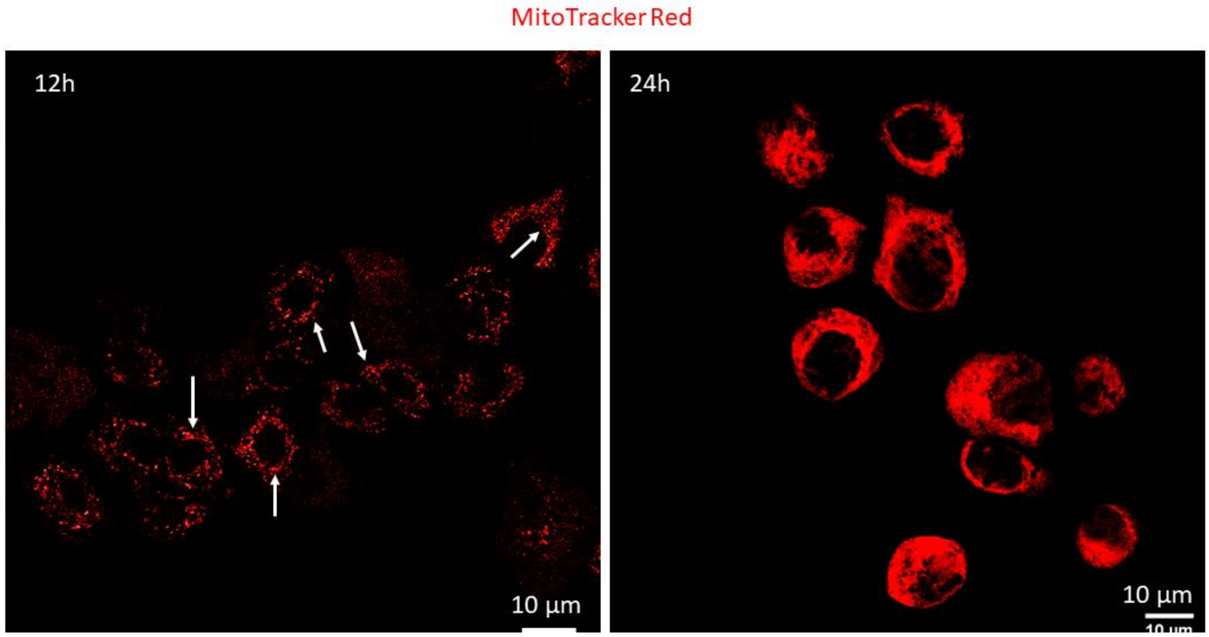

Fig. S10: Confocal microscopy images of A549 cells treated with mt-CNPs followed by staining with red fluorescent MitoTracker Red to visualize the mitochondrial morphology at 12h and 24h. Scale bar = 10 µm.

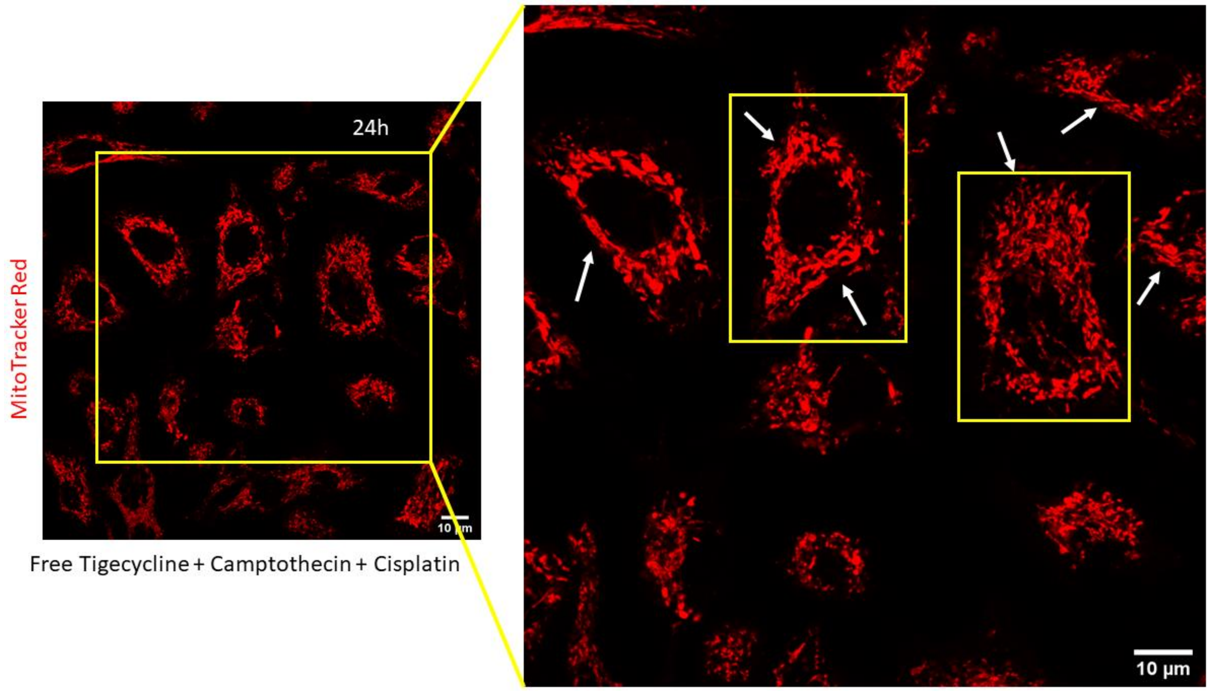

Fig. S11: Confocal microscopy images of A549 cells treated with freed drug combination followed by staining with red fluorescent MitoTracker Red to visualize the mitochondrial morphology at 24h. Scale bar = 10 µm.

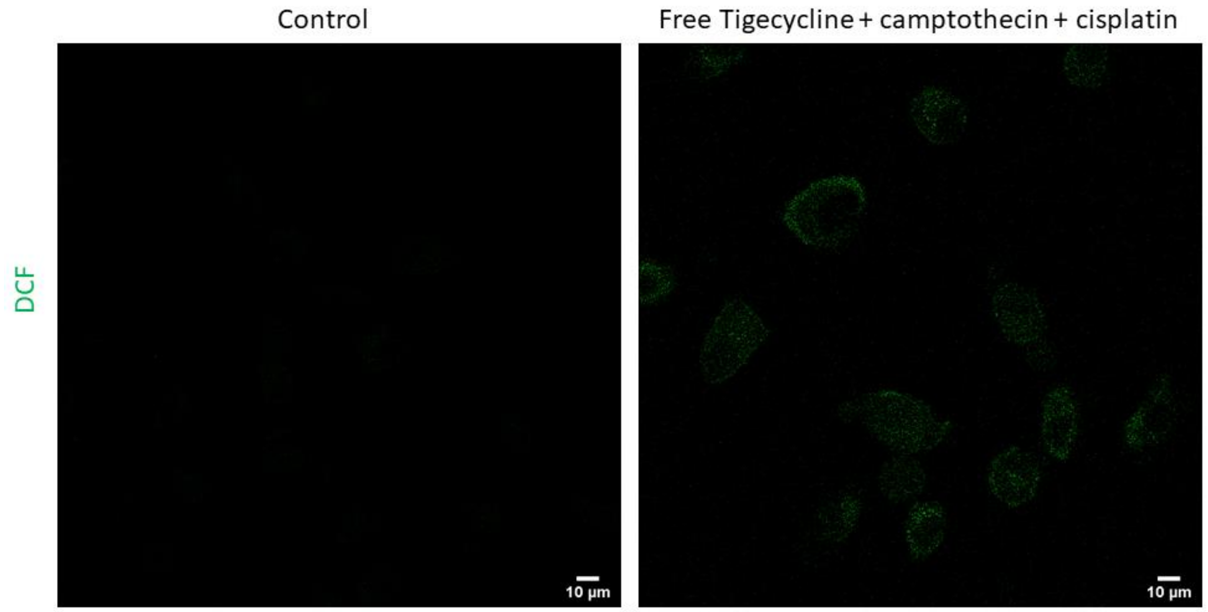

Fig. S12: Confocal microscopy images of A549 cells treated with mt-CNPs followed by staining with H2DCFDA green dye to observe reactive oxygen species (ROS). Scale bar = 10 µm.

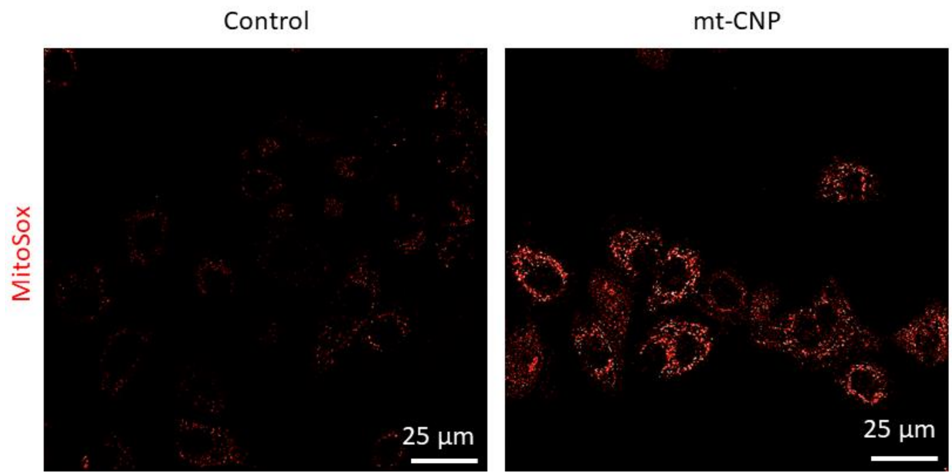

Fig. S13: Confocal microscopy images of A549 cells treated with mt-CNPs followed by staining with MitoSox red dye to observe reactive oxygen species (ROS). Scale bar = 10 µm.

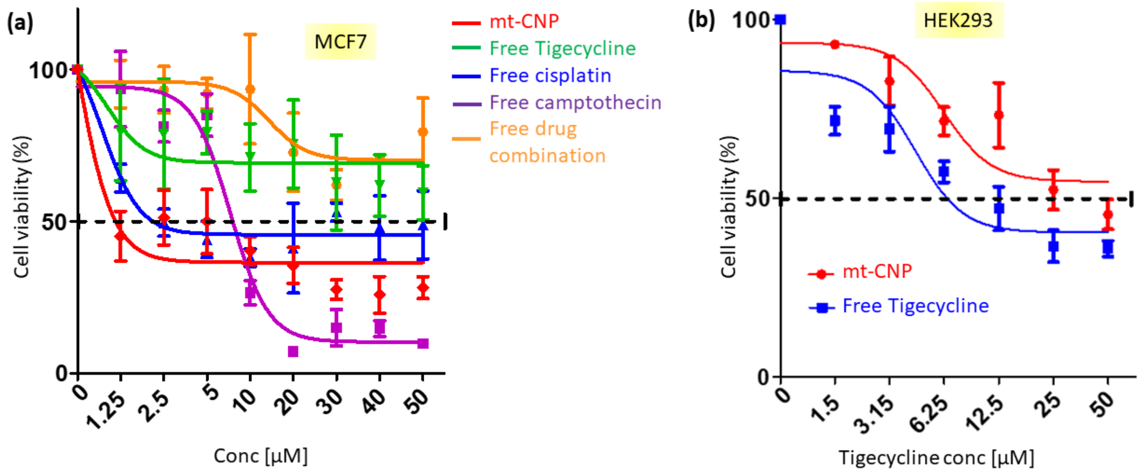

Fig. S14: (a) MTT assay of mt-CNPs in (a) MCF7 and (b) HEK293 cells in a dose dependent manner for 24h post-incubation.

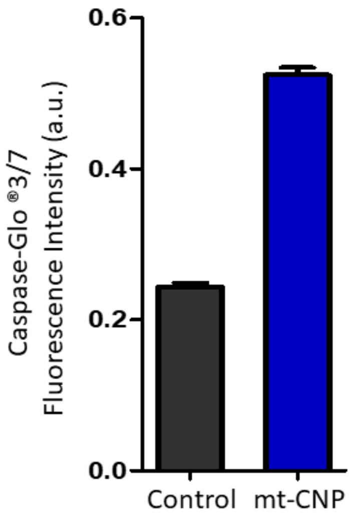

Fig. S15: Quantification of fluorescence intensity from Caspase-Glo ®3/7 assay in A549 cells after treatment with mt-CNPs.
